# Distinct gastroesophageal reflux characteristics in preterm-born infants fed human milk versus formula: insights for clinical practice on outcomes

**DOI:** 10.1038/s41372-025-02421-y

**Published:** 2025-09-17

**Authors:** Erika K. Osborn, Zakia Sultana, Faith Bala, Enas Alshaikh, Sudarshan R. Jadcherla

**Affiliations:** 1 Innovative Infant Feeding Disorders Research Program, Nationwide Children’s Hospital, Columbus, OH USA; 2 Center for Perinatal Research, The Research Institute at Nationwide Children’s Hospital, Columbus, OH USA; 3 Department of Neonatology, Nationwide Children’s Hospital, Columbus, OH USA; 4 Division of Pediatric Gastroenterology, Hepatology, and Nutrition, Department of Pediatrics, The Ohio State University College of Medicine, Columbus, OH USA

**Keywords:** Medical research, Physiology

## Abstract

**Objective:**

To compare the characteristics of gastroesophageal reflux (GER) and clinical outcomes among human milk-fed vs. formula-fed infants evaluated for GER disease (GERD).

**Methods:**

Preterm-born infants (*N* = 316, 30.1 ± 3.4 weeks gestation) referred for 24-h pH-impedance testing at 39.8 ± 1.4 weeks postmenstrual age. GER characteristics, short- and long-term outcomes were compared between the human milk- vs. formula-fed infants.

**Results:**

Human milk-fed infants (vs. formula-fed) had a greater acid reflux index (ARI), and an increased number of acid reflux events, prolonged acid events, and greater proximal acid exposure (*P* < 0.05). There were no differences (*P* > 0.05) in symptoms, acid clearance, bolus clearance, distal baseline impedance, and discharge outcomes. Bayley composite scores at 2 years were distinct (*P* < 0.05) for the distribution (%) of receptive and fine motor skills, favoring human milk-fed infants.

**Conclusions:**

Despite the pathophysiology caused by acid-GER events, human milk properties may provide better adaptation and modulation of neurosensory and neuromotor responses upon esophageal provocation.

## Introduction

Gastroesophageal reflux (GER) is commonly seen among all infants and may be a normal physiological process that improves with maturation and optimal growth [1, 2]. Symptoms can be troublesome during GER when it is attributed to GER disease (GERD) [3, 4]. In convalescing preterm infants anticipating discharge, feeding difficulties amidst troublesome aerodigestive and cardiorespiratory symptoms can be prevalent. Symptom-based GERD definitions can be vague, which means therapies based on symptom-based diagnosis lack objective/mechanistic evidence [5–8]. However, concern for GERD exists as evidenced by a wide variation in prevalence across Neonatal Intensive Care Units (NICU) [9]. Innovative bundled approaches to manage objectively defined GERD using pH-impedance methods have been studied in a randomized controlled manner, and the outcomes were similar in a comparative effectiveness study with infants in both groups showing improvement with maturation and growth regardless of the bundle used [10, 11].

While human milk is considered the gold standard for feeding among preterm infants [12–15], its causal and ameliorative effects in cases of GERD diagnosed using pH-impedance methods remain unclear. Studies have shown that human milk has a faster gastric emptying time compared to formula [16–18]. Although fortification of human milk is recommended in preterm infants to provide adequate nutrients for growth [19], it has been shown to potentially increase the frequency and severity of non-acid GER events [12, 20]. Despite knowing the benefits of fortified human milk, troublesome aerodigestive and cardiorespiratory symptoms purported to be related to GERD result in pharmacological therapies and changes to diet [21–23]. Given this rationale, the objective of this study was to evaluate and compare the characteristics of GER events and clinical outcomes in a large sample of formula-fed versus human milk-fed symptomatic preterm-born infants. Because of the presence of bioactive and neuro-modulatory molecules in human milk, we hypothesized that refluxate characteristics and outcomes would be distinct from formula-fed infants.

## Methods

### Participants and setting

This is a retrospective analysis of prospectively collected data from preterm infants (*N* = 316) with clinical suspicion of GERD who underwent 24-h pH-impedance testing at a single center level 4 NICU. Informed parental consent was obtained before the diagnostic pH-impedance study. Approval for the use of data from these subjects was obtained by the institutional review board, and health insurance portability and accountability guidelines were followed. Data collected included demographic and outcome data from birth, time of the instrumental evaluation, discharge, and follow-up through 26 months’ corrected age, as well as pH-impedance metrics.

This study included preterm infants (born <37 weeks’ gestational age) who underwent pH-impedance studies at our institution between June 2012 and December 2021, with a postmenstrual age (PMA) of 37–42 weeks at the time of the study. Only those subjects who received either all formula or all human milk (including fortified) were included. Subjects were excluded if they received any combination of formula and human milk, or thickened feeds at the time of the pH-impedance study. Additionally, subjects with less than 15 h of pH-impedance data were excluded. Subjects that were on acid-suppressive medications at evaluation were also excluded. All subjects with pH-impedance studies done during this time frame, meeting the inclusion criteria and without exclusion criteria, were analyzed.

### Nutrition and feeding methods

Infants were exclusively fed either infant formula or human milk, which was mostly fortified to ensure adequate nutrient intake. Infants were fed orally (breastfeeding or bottle feeding) or by tube feeding. The specific formula and fortifiers chosen for each infant were determined by the primary care provider and parents based on their individual nutrition and growth requirements [24, 25]. All of the subjects had feeding durations less than 60 min every 2–3 h, and feeding periods were excluded from pH-impedance analysis.

### pH-impedance methods and testing protocol

The 24-h pH-multichannel intraluminal impedance studies were performed using the methodology from published studies [26–31]. Briefly, a pH-impedance catheter with six impedance channels and a pH sensor was connected to a recording device to perform studies. The catheter was calibrated with pH 4.0 and pH 7.0 buffer solutions. The catheter was then placed transnasally by trained nurses above the lower esophageal sphincter and verified by chest x-ray to be at T7 to T8 vertebrae [26]. Trained nursing assistants, blinded to the pH-impedance recordings, documented symptoms and meals in real time at the infant’s bedside using event markers on the recording device for the duration of the entire study. Any events and symptoms documented during mealtimes were not analyzed.

The pH-Impedance analysis was done as described before [23, 26, 28]. Briefly, the pH sensor detected acid reflux events if pH <4 for >5 s, while six impedance sensors detected intraluminal activity from the proximal esophagus to the distal esophagus. These events were visually analyzed by trained investigators using Laborie analysis software (v.9.5, Laborie Medical Technologies, Mississauga, ON, Canada) for spatial, temporal, physical, and chemical properties of GER events [29]. Chemical characteristics detected by the pH sensor included acid reflux index (ARI), which is defined as the duration (%) of esophageal acid exposure. ARI threshold values were followed based on the 2009 North American Society for Pediatric Gastroenterology, Hepatology, and Nutrition clinical practice guidelines, which proposed ARI <3% as normal, 3%–7% as indeterminate, and >7% as abnormal [32]. Other characteristics measured throughout the study included the total number of acid reflux events, the number of acid reflux events >5 min, and acid clearance time. All GER-related data were normalized to a 24-h period. The impedance sensors detected bolus liquid reflux events defined as a retrograde movement with an impedance drop of at least 50% of the baseline originating in the most distal channel and reaching at least the next proximal impedance channel. Mixed refluxes were defined as gas reflux (a rapid rise in impedance >5000 Ω) occurring immediately before or during a liquid reflux. Liquid and mixed events were added to calculate total reflux events. Most proximal ascending events or refluxate reaching the proximal two impedance channels, and bolus clearance time were also analyzed. Esophageal mucosal integrity characteristics were identified using distal baseline impedance [28]. Symptoms were documented in real time during the study and then analyzed by the investigators. A symptom was attributed to a reflux event if any acid and/or bolus reflux event occurred within 2 min prior to the symptom onset. Documented symptoms included arching and irritability/crying, emesis, grunting, flushing, and cardiorespiratory symptoms such as coughing, gagging, sneezing, bradycardia, apnea, and desaturation. Symptom association probability (SAP) was used to identify the statistical relationship between reflux events and symptoms [27, 33]. Any SAP >95% indicated that the association of reflux with the observed symptoms did not occur by chance.

### Statistical analysis

Study participants’ baseline characteristics and clinical outcomes were expressed as mean ± SD or (median ± IQR) of continuous variables and *N* (%) for categorical variables. The two study groups (human milk and formula) were compared using the *t*-test or the Wilcoxon rank-sum test for continuous measures. Categorical data were compared using the chi-squared test.

Depending on the type of variable, logistic regression, analysis of variance (ANOVA), or generalized estimating equations regression was employed to examine the associations or differences between the study groups and each pH-impedance outcome of interest. Repeated measures ANOVA was used to analyze acid clearance time and bolus clearance time, as these variables were measured multiple times within the same participants. Levene’s test was applied to assess the homogeneity of variance. In instances where the assumption of homogeneity was violated, Welch’s ANOVA was conducted as an alternative.

A sub-analysis was done to investigate the association between the two study groups and each of the composite scores for the Bayley scales (cognitive, receptive, and fine motor). We divided composite scores for Bayley into three groups (mid-average function or better, mild impairment/at risk of developmental delay, and moderate to severe impairment) following previous research categorization [34, 35], and a chi-squared test was used to determine the association. This analysis included only participants with documented Bayley’s scores between 18 and 26 months PMA.

All statistical analyses were performed using the SAS 9.4 version. Computer code used to generate results will be made available upon request. Statistical significance was assumed at *P* < 0.05.

## Results

### Participant and clinical outcomes characteristics

There were no significant demographic differences between infants on formula versus human milk either at birth, time of study, or at discharge (Table 1). For this cohort of subjects, we had 8 subjects on unfortified human milk, 23 subjects fortified with human milk fortifier, 11 subjects fortified with hypoallergenic formulas, 69 subjects fortified with preterm formula powder, and 4 subjects fortified with term formula powder. At discharge, 15 subjects were receiving full unfortified human milk.

**Table 1:** Infant characteristics at birth, evaluation, and discharge comparing differences between human milk- versus formula-fed infants.

Characteristics	Formula (*N* = 201)	Human Milk (*N* = 115)	*P* Value
**AT BIRTH**			
Gender (male), *n* (%)	87 (43)	58 (50)	0.22
Gestational age, weeks	30.3 ± 3.5	29.7 ± 3.2	0.16
Weight, grams	1566.5 ± 852.1	1389.8 ± 608.4	0.05
Length, cm	39.4 ± 5.8	27.1 ± 3.4	0.37
Head circumference, cm	27.6 ± 3.8	38.81 ± 5.2	0.22
**AT EVALUATION**			
Postmenstrual age, weeks	39.8 ± 1.4	39.7 ± 1.5	0.80
Weight, grams	3118.7 ± 596.7	3087.5 ± 561.7	0.65
Length, cm	47.5 ± 3.0	47.6 ± 3.2	0.76
Head circumference, cm	33.5 ± 1.8	33.8 ± 1.7	0.09
Feeding (tube: transition: oral), %	6:36:58	3: 49: 48	0.09
Any respiratory support, *n* (%)	62 (31)	46 (40)	0.10
Bronchopulmonary dysplasia, *n* (%)	76 (38)	54 (47)	0.22
Neurological disease, *n* (%)	71 (35)	34 (30)	0.30
Congenital anomalies, *n* (%)	15 (7)	12 (10)	0.36
**AT DISCHARGE**			
Postmenstrual age, weeks	43.4 ± 4.0	43.6 ± 4.6	0.73
Length of hospital stay, days	91.8 ± 42.6	96.9 ± 43.5	0.31
Weight, grams	3797.8 ± 921	3839.0 ± 961.6	0.71
Length, cm	50.8 ± 3.9	50.9 ± 3.7	0.75
Head circumference, cm	35.3 ± 2.2	35.7 ± 2.0	0.11
Feeding (transition: oral), %	10: 90	14: 86	0.29
Any respiratory support, *n* (%)	52 (26)	41 (36)	0.07

Data presented as mean ± SD, %, or *n* (%).

**Fig. 1:**
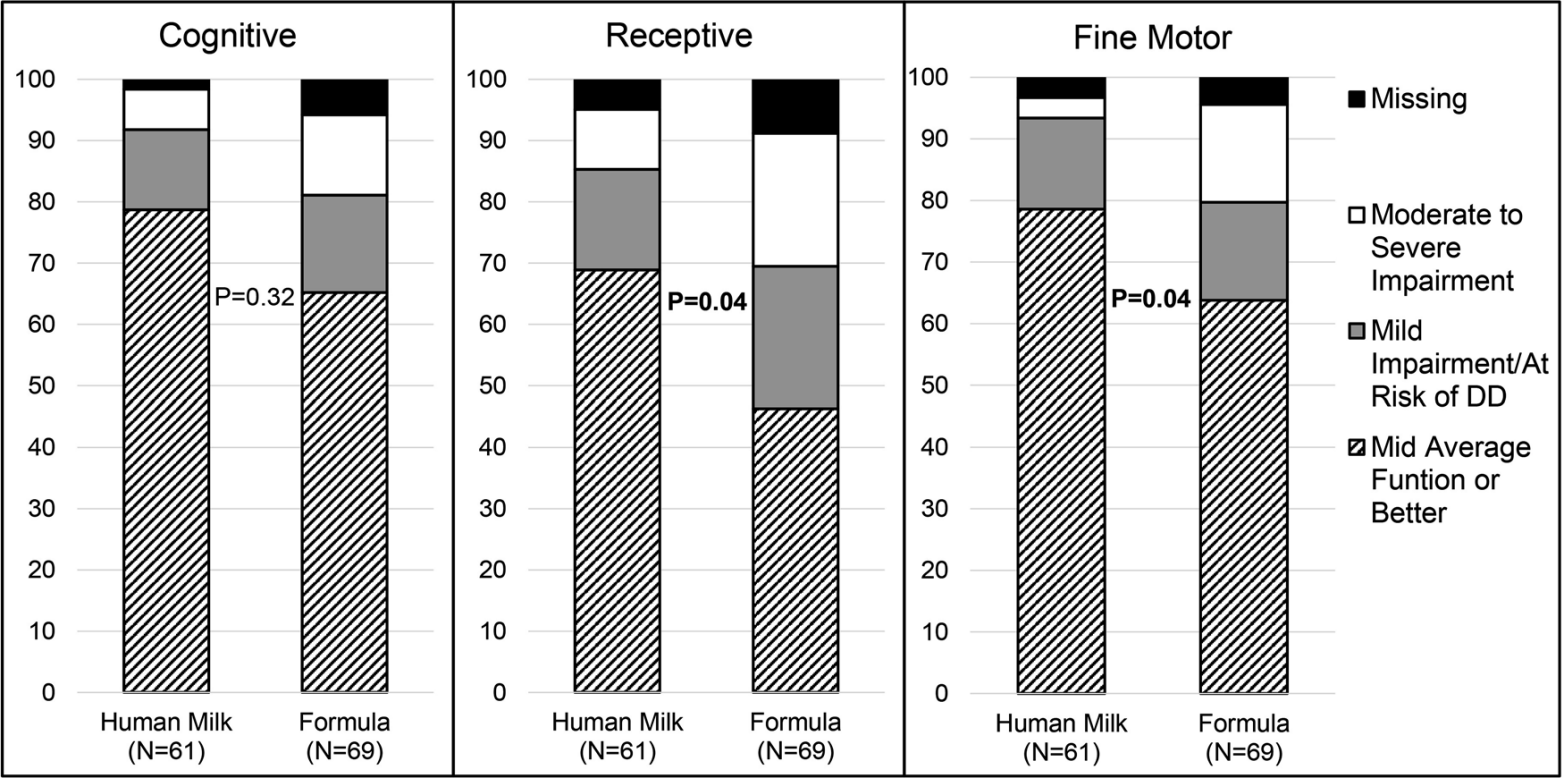
Bayley composite scores category based on diet at the time of the study. The composite score for Bayley was calculated for cognitive, receptive, and motor scales. The scores are interpreted as moderate to severe impairment, mild impairment, and mid-average function or better. There was no significant difference in the cognitive scale between human milk-fed and formula-fed infants. Human milk-fed infants had significantly higher mid-average function or better scores for receptive and fine motor scales compared to formula-fed infants.

Discharge outcomes were similar; however, Bayley composite scores in those who completed an assessment between 18 and 26 months corrected age (Human Milk *N* = 61, Formula *N* = 69) were distinct. When divided into categories, those subjects who received all human milk had significantly better receptive and fine motor composite scores (both *P* < 0.05) (Fig. 1).

### Effect of human milk and formula on pH-impedance analysis

**Fig. 2:**
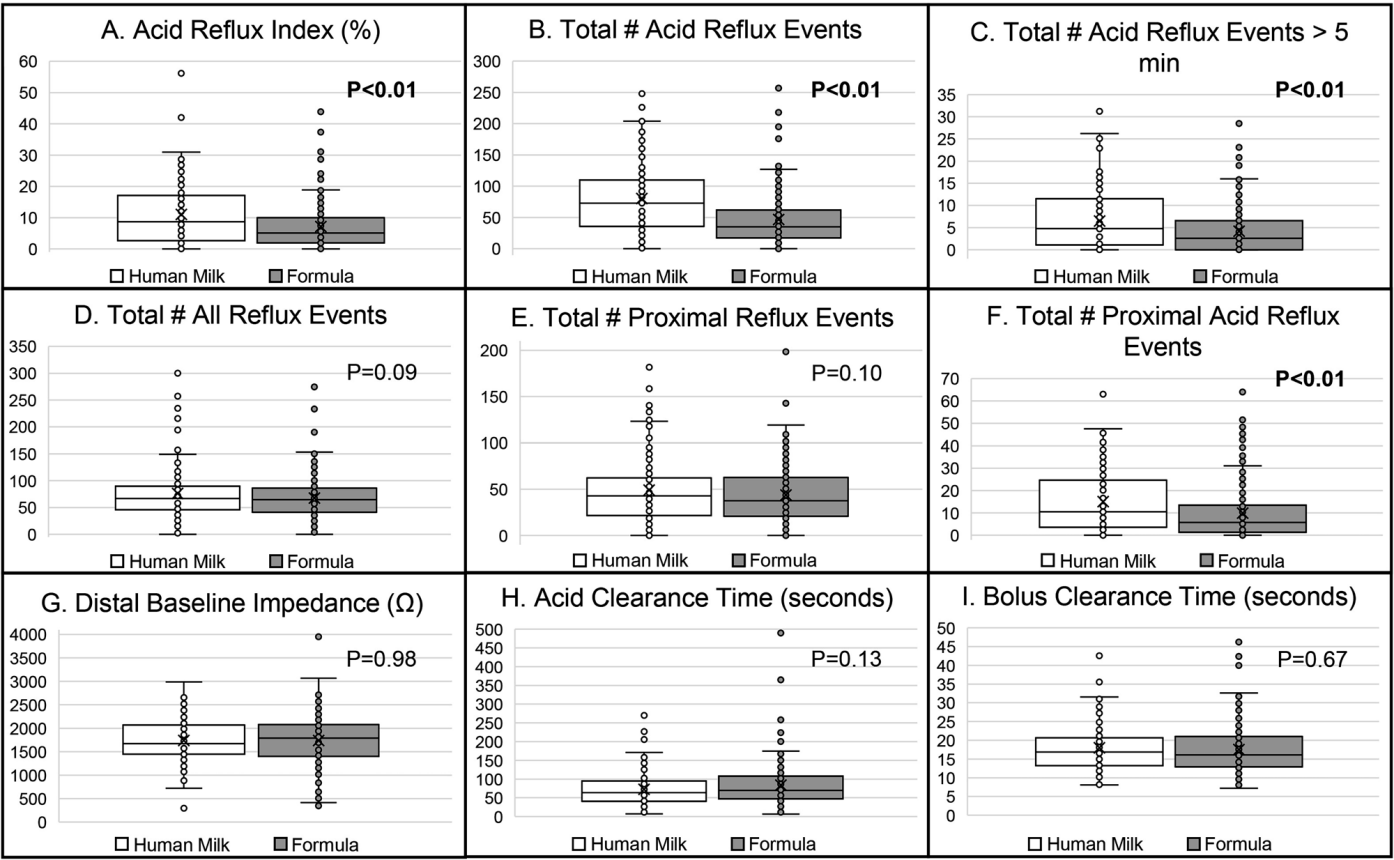
Comparison of pH-impedance results. The star represents the mean, and the line represents the median. All total # results were standardized to a 24-h period. Infants who were fed human milk had higher **A** acid reflux index, **B** total # of acid reflux events, **C** total # of acid reflux events >5 min, and **F** total # of proximal acid reflux events (*P* < 0.05). However, **D** total # of all reflux events, **E** total # of proximal reflux events, **G** distal baseline impedance, **H** acid clearance time, and **I** bolus clearance time were similar across human milk-fed and formula-fed infants.

**Fig. 3:**
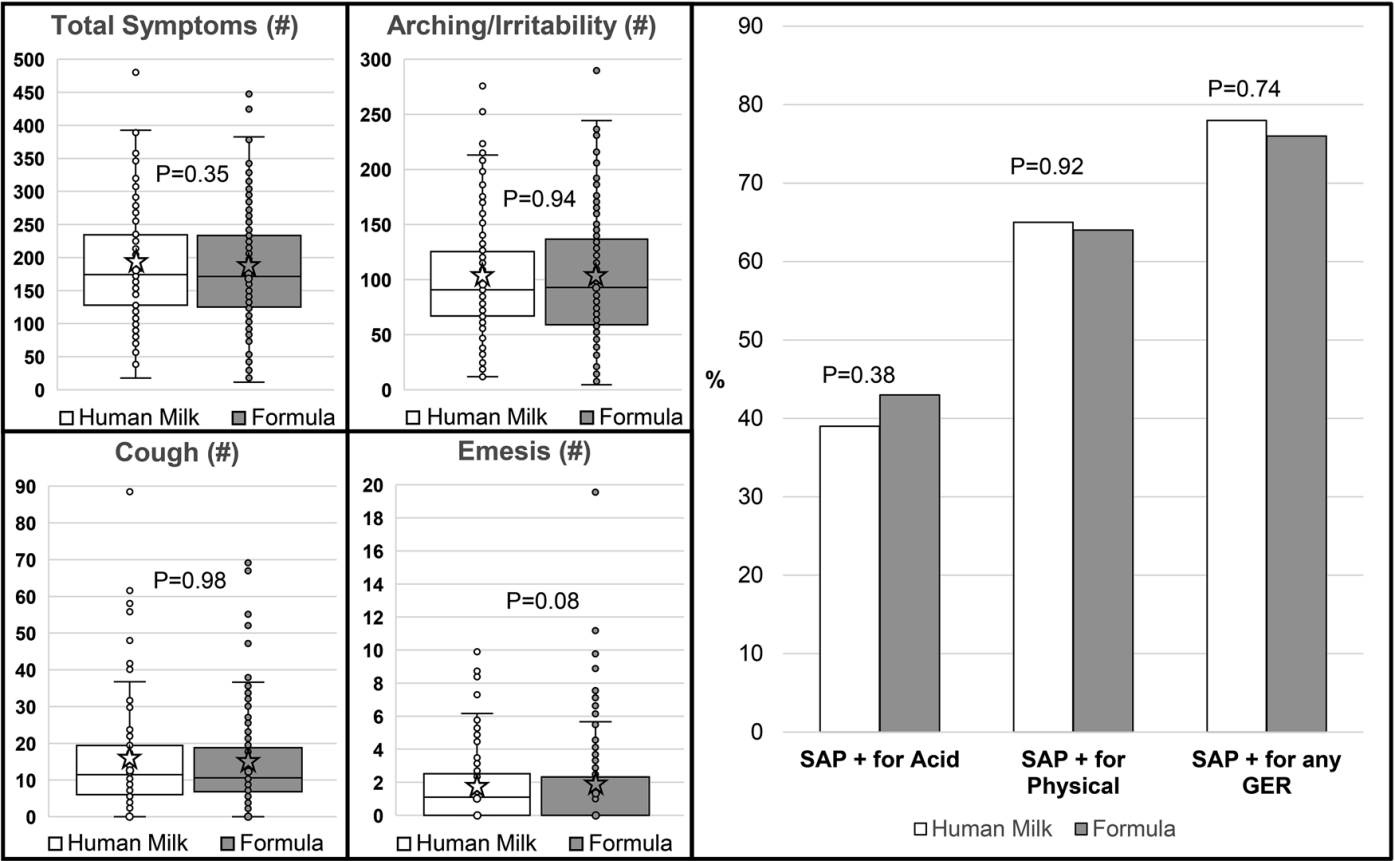
Comparison of symptoms among human milk-fed and formula-fed infants. SAP symptom association probability. All symptoms were standardized to a 24-h period, and the star and line represent the mean and median, respectively. There were no significant differences in arching/irritability, cough, emesis, and total symptoms between human milk-fed versus formula-fed infants. SAP > 95% for acid events (as detected by pH) was classified as positive SAP for acid, and SAP > 95% for bolus events (as detected by impedance) was classified as SAP positive for bolus, and SAP > 95% for both acid and bolus was classified as SAP positive for any GER events. No significant differences were observed in SAP positive for acid, bolus, or any GER between human milk-fed and formula-fed infants.

Analyses of refluxate characteristics between those fed human milk versus formula during the pH-impedance study are shown in Fig. 2A–I. Subjects fed human milk during the study had significantly higher esophageal acid exposure in comparison to formula, specifically ARI, 11.0 ± 9.9 vs 7.1 ± 7.0, respectively (Fig. 2A), number of acid reflux events, 80.4 ± 57.7 vs 46.7 ± 42.3, respectively (Fig. 2B), number of acid reflux events >5 min, 6.5 ± 6.6 vs 4.2 ± 4.7, respectively (Fig. 2C), and proximal acid events, 20.2 ± 1.4 vs 12.9 ± 1.1, respectively (Fig. 2D). ARI thresholds (<3%, 3%–7%, >7%) for infants fed human milk were 27:14: 59 (%), respectively, vs formula-fed infants were 35:23:42 (%), respectively, *P* = 0.01. There were 68 (59%) infants fed human milk with ARI > 7% vs 84 (42%) of formula-fed infants with ARI > 7%, *P* = 0.003. Additionally, infants fed human milk had a significantly higher number of reflux events >70 vs formula-fed infants, 86 (75%) vs 97 (48%), respectively, *P* = 0.0001. There were no significant differences noted in acid or bolus clearance time, distal baseline impedance, total number of symptoms, or specific symptoms of arching/irritability, coughing, or emesis. Comparison of SAP (Fig. 3) showed no difference in SAP for acid reflux, bolus reflux, or any reflux. Despite the human milk group having higher acid exposure, there was no significant difference between the number of infants who were prescribed and received acid-suppressive medications before discharge (human milk = 70%, formula = 61%, *P* = 0.09).

## Discussion

Convalescing infants frequently experience GER events accompanied by distressing symptoms (GERD), which poses a diagnostic and therapeutic conundrum to the providers. Objective diagnostic pH-impedance criteria [26, 36] were used to critically examine the influence of human milk versus infant formula on the biomarkers of GERD and clinical outcomes in this study. Given that the demographic and clinical characteristics were similar in the formula-fed versus human milk-fed groups (Table 1), the findings are significant to indicate the nutrient-type effects on the studied biomarkers. The salient findings are as follows: Infants fed human milk had increased ARI, increased acid reflux events, increased number of prolonged acid reflux events, and increased number of proximal acid reflux events. However, there were no differences in overall reflux events (including non-acid events), distal baseline impedance (mucosal integrity marker), total proximal events (all retrograde movements), acid clearance time (neutralization of esophageal acidity exposure marker), bolus clearance time (esophageal peristalsis), or symptoms. While other specific symptoms were documented and analyzed, we chose to report only arching and irritability/crying, cough, and emesis, as these were the most prevalent symptoms and demonstrated esophageal sensitivity to acid or bolus based on prior findings [22, 27]. Total symptoms are reported to include other specific symptoms. The study findings suggest that human milk increases esophageal acid exposure, but these acid reflux events are not contributing to any greater mucosal impairment, clearance times, or increase in troublesome symptoms (compared to formula-fed infants). Furthermore, superior neurodevelopmental outcomes among infants fed human milk were noted around 2 years of age.

The nutrient composition of different milk types may have contributed to these observations. In a prior study done in full-term born infants using pH-metry only [37], the human milk-fed group showed significantly lower median pH values, but the duration of acid clearance was also considerably shorter, suggesting more frequent but shorter duration of acid reflux events. In another study, an inverse relationship between the protein content of human milk, the occurrence of acid GER events, and ARI was observed [20]. Moreover, human milk oligosaccharides can decrease infection by acidifying the gut mucosa, binding to pathogens, and repressing inflammation at the mucosa level [38, 39]. In our study, the mucosal integrity marker (baseline impedance) was within normal ranges. Human milk also contains digestive enzymes (lipase, amylase, and α1-antitrypsin), growth factors, and lactoferrin, which support digestive function and intestinal development [15, 40, 41]. Furthermore, human milk contains epidermal growth factor, vascular endothelial growth factor, and certain cytokines (interleukin 10, interleukin 1 receptor antagonist, and tumor necrosis factor receptor I and II), which have been shown to have anti-inflammatory properties [38, 42].

Additionally, the pH of human milk may be more acidic due to maternal diet, fortification status, and storage duration [43]. Research suggests that the pH of human milk becomes more acidic over the postpartum period and with prolonged refrigeration [43]. Preterm infants are often fed human milk that was refrigerated, as direct breastfeeding is often challenging in the NICU. In addition, both human milk and infant formula are potent buffers of gastric acidity, and the pH of gastric content is raised (more towards alkalinity) for 1–2 h after milk feeds [44]. Gastric emptying is faster in human milk-fed infants than in formula milk-fed infants [17, 45]. It is possible that faster gastric emptying after human milk feeding reduces the duration of gastric acid buffering, leading to rapid reacidification of the stomach and resulting in more acidic GER. However, research has not established a relationship between the rate of gastric emptying and any indices of GER in infants [46].

Considering all these research studies in the context of the current study findings, the increased esophageal acid exposure associated with human milk feeds may have a protective role by reducing pathogens, promoting healing, and facilitating the repair and regeneration of the aerodigestive mucosa. Human milk is connected to acid exposure, healing, and repair of GI mucosa, but how this is all related to superior Bayley scores cannot be ascertained by the clinical nature of this retrospective study. Future studies may explore these observations in prospective studies or with larger datasets.

On a different note, the upstream esophageal motility, aerodigestive protective mechanisms, and downstream gastrointestinal motility are influenced by vagal nerve maturation and cross-talk between the participatory systems [47]. These reflexes, particularly the gastrointestinal and gastro-esophageal reflexes, are modulated by sensory stimuli, which play a role in evoking motility and adaptive responses. Physical or chemical properties of stimuli provided may influence responses, or varying maturational changes may alter adaptation to stimuli [11, 48]. While clinical and maturational characteristics were similar at birth, evaluation, and discharge, it is possible that infants fed human milk have appropriate bolus clearance (peristalsis) and adaptational mechanisms despite having greater acid GER indices. These findings may be due to vagal nerve-dependent modulatory effects on sensory-motor events favoring aerodigestive clearance and adaptation.

### Strengths and limitations

Existing studies examining the effects of formula versus human milk on GER using pH-impedance monitoring are relatively limited [37]. By utilizing pH-impedance with symptom correlation, this study is particularly valuable for understanding the significance of spatial, temporal, physical, and chemical characteristics of reflux events in relation to milk type [29, 36]. While the neurodevelopmental benefits of human milk feeding are well established [49], our findings of superior neurodevelopmental scores despite higher ARI based on objectively defined GERD using pH-impedance criteria are novel. Our data is from a 24-h pH-impedance study, and there could be feed-to-feed variability in either group that was not analyzable due to potential variations in feeding techniques or positioning. However, our previous study demonstrated that a feeding modification bundle (volumes fed, intra- and postprandial body positions, and feeding durations) did not show any differences in GER symptoms or aerodigestive reflexes [10, 11]. Rather, maturational changes appeared to play a more crucial role in the improvement of GERD, suggesting nutrition and growth are central to overall improvement. In another recent study, we found that while partially oral-fed (oral+ nasogastric tube) infants had lower ARI, exclusively oral-fed infants with increased acid exposure had better oral intake and shorter hospitalizations [50]. However, the reflux parameters explored in that study were limited. Future studies are needed to further examine the difference between tube-fed and exclusively oral-fed infants.

Another limitation of this retrospective study was the variability in fortification practices. Both formula-fed and human milk-fed groups received diverse fortification types and amounts based on infants’ nutritional needs, which we were unable to standardize or fully analyze due to the retrospective nature of the data. Consequently, the specific composition of different formulas and the caloric density of fortifiers remain unknown. It is also important to note that our long-term outcome was limited only to Bayley’s scores, and it is likely that data on sociodemographic, level of education, and economic variables may have impacted scores.

## Conclusion

Compared to the formula-fed cohort, the human milk-fed group had significantly greater acid-related esophageal motility events. Despite this, both groups exhibited similar symptoms, mucosal inflammatory markers, and discharge outcomes. Notably, the human milk-fed group demonstrated superior long-term neurodevelopmental outcomes. The pathophysiology caused by acid GER events may be different, but the human milk properties may have provided better adaptation and modulation of neurosensory and neuromotor responses upon esophageal provocation. The modulatory and linking effects of human milk on gastrointestinal and neurodevelopmental functions may contribute to the studied outcomes. It is possible that a higher ARI in the human milk-fed group may not be pathological in the context of nutrient intake, especially when not associated with increased mucosal impairment, troublesome symptoms, oral feeding difficulties, or motility disturbances. Further investigation is needed to determine the significance of these observations.

## Data Availability

The data that support the findings of this study are available upon reasonable request from the corresponding author. The data are not publicly available due to privacy and ethical restrictions.

## References

[1] Jadcherla SR, Rudolph C. Gastroesophageal reflux in the preterm neonate. Neoreviews. 2005;6:e87–98.

[2] Corvaglia L, Martini S, Aceti A, Arcuri S, Rossini R, Faldella G. Nonpharmacological management of gastroesophageal reflux in preterm infants. Biomed Res Int. 2013;2013:141967. 24073393 10.1155/2013/141967 PMC3773993

[3] Rosen R, Vandenplas Y, Singendonk M, Cabana M, DiLorenzo C, Gottrand F, et al. Pediatric Gastroesophageal Reflux Clinical Practice Guidelines: joint recommendations of the North American Society for Pediatric Gastroenterology, Hepatology, and Nutrition and the European Society for Pediatric Gastroenterology, Hepatology, and Nutrition. J Pediatr Gastroenterol Nutr. 2018;66:516–54. 29470322 10.1097/MPG.0000000000001889 PMC5958910

[4] Sherman PM, Hassall E, Fagundes-Neto U, Gold BD, Kato S, Koletzko S, et al. A global, evidence-based consensus on the definition of gastroesophageal reflux disease in the pediatric population. Am J Gastroenterol. 2009;104:1278–95. 19352345 10.1038/ajg.2009.129

[5] Bellodas Sanchez J, Jadcherla SR. Gastroesophageal reflux disease in neonates: facts and figures. Neoreviews. 2021;22:e104–17. 33526640 10.1542/neo.22-2-e104

[6] Sultana Z, Hasenstab KA, Moore RK, Osborn EK, Yildiz VO, Wei L, et al. Symptom scores and pH-impedance: secondary analysis of a randomized controlled trial in infants treated for gastroesophageal reflux. Gastro Hep Adv. 2022;1:869–81. 36310566 10.1016/j.gastha.2022.06.004 PMC9615096

[7] Eichenwald EC, Committee on Fetus and Newborn. Diagnosis and management of gastroesophageal reflux in preterm infants. Pediatrics. 2018;142:e20181061. 10.1542/peds.2018-1061 29915158

[8] Gonzalez Ayerbe JI, Hauser B, Salvatore S, Vandenplas Y. Diagnosis and management of gastroesophageal reflux disease in infants and children: from guidelines to clinical practice. Pediatr Gastroenterol Hepatol Nutr. 2019;22:107–21. 30899687 10.5223/pghn.2019.22.2.107 PMC6416385

[9] Jadcherla SR, Slaughter JL, Stenger MR, Klebanoff M, Kelleher K, Gardner W. Practice variance, prevalence, and economic burden of premature infants diagnosed with GERD. Hosp Pediatr. 2013;3:335–41. 24435191 10.1542/hpeds.2013-0036 PMC4075760

[10] Jadcherla SR, Hasenstab KA, Wei L, Osborn EK, Viswanathan S, Gulati IK, et al. Role of feeding strategy bundle with acid-suppressive therapy in infants with esophageal acid reflux exposure: a randomized controlled trial. Pediatr Res. 2021;89:645–52. 32380509 10.1038/s41390-020-0932-4 PMC7647955

[11] Jadcherla SR, Hasenstab KA, Gulati IK, Helmick R, Ipek H, Yildiz V, et al. Impact of feeding strategies with acid suppression on esophageal reflexes in human neonates with gastroesophageal reflux disease: a single-blinded randomized clinical trial. Clin Transl Gastroenterol. 2020;11:e00249. 33259163 10.14309/ctg.0000000000000249 PMC7643906

[12] Meek JY, Noble L. Section on Breastfeeding. Policy Statement: breastfeeding and the use of human milk. Pediatrics. 2022;150:e2022057988. 10.1542/peds.2022-057988 35921640

[13] Parker MG, Stellwagen LM, Noble L, Kim JH, Poindexter BB, Puopolo KM, et al. Promoting human milk and breastfeeding for the very low birth weight infant. Pediatrics. 2021;148:e2021054272. 10.1542/peds.2021-054272 34635582

[14] Hair AB, Peluso AM, Hawthorne KM, Perez J, Smith DP, Khan JY, et al. Beyond necrotizing enterocolitis prevention: improving outcomes with an exclusive human milk-based diet. Breastfeed Med. 2016;11:70–4. 26789484 10.1089/bfm.2015.0134 PMC4782036

[15] Ballard O, Morrow AL. Human milk composition: nutrients and bioactive factors. Pediatr Clin North Am. 2013;60:49–74. 23178060 10.1016/j.pcl.2012.10.002 PMC3586783

[16] Meyer R, Foong RX, Thapar N, Kritas S, Shah N. Systematic review of the impact of feed protein type and degree of hydrolysis on gastric emptying in children. BMC Gastroenterol. 2015;15:137. 26472544 10.1186/s12876-015-0369-0 PMC4608328

[17] Cavell B. Gastric emptying in infants fed human milk or infant formula. Acta Paediatr Scand. 1981;70:639–41. 7324911

[18] Ewer AK, Durbin GM, Morgan ME, Booth IW. Gastric emptying in preterm infants. Arch Dis Child Fetal Neonatal Ed. 1994;71:F24–7. 8092865 10.1136/fn.71.1.f24 PMC1061063

[19] Bergner EM, Taylor SN, Gollins LA, Hair AB. Human milk fortification: a practical analysis of current evidence. Clin Perinatol. 2022;49:447–60. 35659096 10.1016/j.clp.2022.02.010

[20] Aceti A, Corvaglia L, Paoletti V, Mariani E, Ancora G, Galletti S, et al. Protein content and fortification of human milk influence gastroesophageal reflux in preterm infants. J Pediatr Gastroenterol Nutr. 2009;49:613–8. 19633575 10.1097/MPG.0b013e31819c0ce5

[21] Slaughter JL, Stenger MR, Reagan PB, Jadcherla SR. Neonatal histamine-2 receptor antagonist and proton pump inhibitor treatment at United States children’s hospitals. J Pediatr. 2016;174:63–70.e3. 27131401 10.1016/j.jpeds.2016.03.059 PMC4925209

[22] Njeh M, Helmick R, Alshaikh E, Marcano K, Alexander A, Osborn E, et al. The irritable infant in the neonatal intensive care unit: risk factors and biomarkers of gastroesophageal reflux disease. J Pediatr. 2024;264:113760. 37777170 10.1016/j.jpeds.2023.113760 PMC13431874

[23] Sultana Z, O Yildiz V, Jadcherla SR. Characteristics of esophageal refluxate and symptoms in infants compared between pre-treatment and on treatment with proton pump inhibitors. J Perinatol. 2024;44:87–93. 37980392 10.1038/s41372-023-01825-y PMC13455182

[24] Osborn EK, Jadcherla SR. Developing a quality improvement feeding program for NICU patients. Neoreviews. 2022;23:e23–35. 34970663 10.1542/neo.23-1-e23

[25] Jadcherla SR, Dail J, Malkar MB, McClead R, Kelleher K, Nelin L. Impact of process optimization and quality improvement measures on neonatal feeding outcomes at an all-referral neonatal intensive care unit. J Parenter Enter Nutr. 2016;40:646–55. 10.1177/0148607115571667 25733339

[26] Sivalingam M, Sitaram S, Hasenstab KA, Wei L, Woodley FW, Jadcherla SR. Effects of esophageal acidification on troublesome symptoms: an approach to characterize true acid GERD in dysphagic neonates. Dysphagia. 2017;32:509–19. 28365873 10.1007/s00455-017-9792-4 PMC5733803

[27] Jadcherla SR, Sultana Z, Hasenstab-Kenney KA, Prabhakar V, Gulati IK, Di Lorenzo C. Differentiating esophageal sensitivity phenotypes using pH-impedance in intensive care unit infants referred for gastroesophageal reflux symptoms. Pediatr Res. 2021;89:636–44. 32375162 10.1038/s41390-020-0930-6 PMC7644596

[28] Jadcherla SR, Hanandeh N, Hasenstab KA, Nawaz S. Differentiation of esophageal pH-impedance characteristics classified by the mucosal integrity marker in human neonates. Pediatr Res. 2019;85:355–60. 30467343 10.1038/s41390-018-0237-z PMC6377827

[29] Jadcherla SR, Gupta A, Fernandez S, Nelin LD, Castile R, Gest AL, et al. Spatiotemporal characteristics of acid refluxate and relationship to symptoms in premature and term infants with chronic lung disease. Am J Gastroenterol. 2008;103:720–8. 18341491 10.1111/j.1572-0241.2007.01748.x

[30] Lopez-Alonso M, Moya MJ, Cabo JA, Ribas J, del Carmen Macias M, Silny J, et al. Twenty-four-hour esophageal impedance-pH monitoring in healthy preterm neonates: rate and characteristics of acid, weakly acidic, and weakly alkaline gastroesophageal reflux. Pediatrics. 2006;118:e299–308. 16831894 10.1542/peds.2005-3140

[31] Wenzl TG, Benninga MA, Loots CM, Salvatore S, Vandenplas Y, ESPGHAN EURO-PIG Working Group. Indications, methodology, and interpretation of combined esophageal impedance-pH monitoring in children: ESPGHAN EURO-PIG standard protocol. J Pediatr Gastroenterol Nutr. 2012;55:230–4. 10.1097/MPG.0b013e3182592b65 22711055

[32] Vandenplas Y, Rudolph CD, Di Lorenzo C, Hassall E, Liptak G, Mazur L, et al. Pediatric gastroesophageal reflux clinical practice guidelines: joint recommendations of the North American Society for Pediatric Gastroenterology, Hepatology, and Nutrition (NASPGHAN) and the European Society for Pediatric Gastroenterology, Hepatology, and Nutrition (ESPGHAN). J Pediatr Gastroenterol Nutr. 2009;49:498–547. 19745761 10.1097/MPG.0b013e3181b7f563

[33] Weusten BL, Roelofs JM, Akkermans LM, Van Berge-Henegouwen GP, Smout AJ. The symptom-association probability: an improved method for symptom analysis of 24-hour esophageal pH data. Gastroenterology. 1994;107:1741–5. 7958686 10.1016/0016-5085(94)90815-x

[34] Del Rosario C, Slevin M, Molloy EJ, Quigley J, Nixon E. How to use the Bayley scales of infant and toddler development. Arch Dis Child Educ Pract Ed. 2021;106:108–12. 32859738 10.1136/archdischild-2020-319063

[35] Jadcherla SR, Khot T, Moore R, Malkar M, Gulati IK, Slaughter JL. Feeding methods at discharge predict long-term feeding and neurodevelopmental outcomes in preterm infants referred for gastrostomy evaluation. J Pediatr. 2017;181:125–30.e1. 27939123 10.1016/j.jpeds.2016.10.065 PMC5724518

[36] Njeh M, Sultana Z, Plumb T, Alshaikh E, Jadcherla SR. Comparison of direct effects of rice-thickened formula vs routine feeds on symptoms and gastroesophageal reflux indices: a crossover cohort study. J Parenter Enter Nutr. 2024;48:64–73. 10.1002/jpen.2566 37850573

[37] Heacock HJ, Jeffery HE, Baker JL, Page M. Influence of breast versus formula milk on physiological gastroesophageal reflux in healthy, newborn infants. J Pediatr Gastroenterol Nutr. 1992;14:41–6. 1573512 10.1097/00005176-199201000-00009

[38] Thai JD, Gregory KE. Bioactive factors in human breast milk attenuate intestinal inflammation during early life. Nutrients. 2020;12:581. 10.3390/nu12020581 PMC7071406 32102231

[39] Morrow AL, Ruiz-Palacios GM, Altaye M, Jiang X, Guerrero ML, Meinzen-Derr JK, et al. Human milk oligosaccharides are associated with protection against diarrhea in breast-fed infants. J Pediatr. 2004;145:297–303. 15343178 10.1016/j.jpeds.2004.04.054

[40] Haschke F, Haiden N, Thakkar SK. Nutritive and bioactive proteins in breastmilk. Ann Nutr Metab. 2016;69:17–26. 28103610 10.1159/000452820

[41] Taylor SN. Solely human milk diets for preterm infants. Semin Perinatol. 2019;43:151158. 31301819 10.1053/j.semperi.2019.06.006

[42] Chatterton DE, Nguyen DN, Bering SB, Sangild PT. Anti-inflammatory mechanisms of bioactive milk proteins in the intestine of newborns. Int J Biochem Cell Biol. 2013;45:1730–47. 23660296 10.1016/j.biocel.2013.04.028

[43] Filatava EJ, Shelly CE, Overton NE, Gregas M, Glynn R, Gregory KE. Human milk pH is associated with fortification, postpartum day, and maternal dietary intake in preterm mother-infant dyads. J Perinatol. 2023;43:60–7. 35978105 10.1038/s41372-022-01492-5 PMC9840648

[44] Mitchell DJ, McClure BG, Tubman TR. Simultaneous monitoring of gastric and oesophageal pH reveals limitations of conventional oesophageal pH monitoring in milk fed infants. Arch Dis Child. 2001;84:273–6. 11207184 10.1136/adc.84.3.273 PMC1718697

[45] Ewer AK, Yu VY. Gastric emptying in pre-term infants: the effect of breast milk fortifier. Acta Paediatr. 1996;85:1112–5. 8888928 10.1111/j.1651-2227.1996.tb14227.x

[46] Ewer AK, Durbin GM, Morgan ME, Booth IW. Gastric emptying and gastro-oesophageal reflux in preterm infants. Arch Dis Child Fetal Neonatal Ed. 1996;75:F117–21. 8949695 10.1136/fn.75.2.f117 PMC1061175

[47] Jadcherla SR, Chan CY, Fernandez S, Splaingard M. Maturation of upstream and downstream esophageal reflexes in human premature neonates: the role of sleep and awake states. Am J Physiol Gastrointest Liver Physiol. 2013;305:G649–58. 24008357 10.1152/ajpgi.00002.2013 PMC3840236

[48] Gupta A, Gulati P, Kim W, Fernandez S, Shaker R, Jadcherla SR. Effect of postnatal maturation on the mechanisms of esophageal propulsion in preterm human neonates: primary and secondary peristalsis. Am J Gastroenterol. 2009;104:411–9. 19174814 10.1038/ajg.2008.32 PMC3796765

[49] Berger PK, Ong ML, Bode L, Belfort MB. Human milk oligosaccharides and infant neurodevelopment: a narrative review. Nutrients. 2023;15:719. 10.3390/nu15030719 PMC9918893 36771425

[50] Alexander A, Helmick R, Plumb T, Alshaikh E, Jadcherla SR. Characterizing biomarkers of continuous peristalsis and bolus transit during oral feeding in infants at pH-impedance evaluation: clinical and research implications. J Pediatr. 2024;274:114154. 38897379 10.1016/j.jpeds.2024.114154

